# Early Recognition and Treatment of Relapsing Polychondritis

**DOI:** 10.7759/cureus.21463

**Published:** 2022-01-20

**Authors:** Toyoshi Yanagihara, Migiwa Ohgushi, Taro Setoguchi, Naruhiko Ogo, Yu Inutsuka, Haruna Fujiwara, Tatsuma Asoh, Syunya Sunami, Reiko Yoneda, Takashige Maeyama

**Affiliations:** 1 Department of Respiratory Medicine, Hamanomachi Hospital, Fukuoka, JPN; 2 Department of General Medicine, Hamanomachi Hospital, Fukuoka, JPN; 3 Department of Radiology, Hamanomachi Hospital, Fukuoka, JPN; 4 Department of Pathology, Hamanomachi Hospital, Fukuoka, JPN

**Keywords:** anti-type 2 collagen antibody, autoimmune disease, ear swelling, c-reactive protein, relapsing polychondritis

## Abstract

We describe the case of a 60-year-old Japanese man with relapsing polychondritis (RP). The patient was referred to Hamanomachi Hospital due to mild elevation of C-reactive protein and mild anemia on medical checkup without any symptoms. Body CT imaging showed thickened tracheal and bronchial walls with no active lesions in the lung. Precise physical examination revealed swelling in both ears. Bronchoscopy revealed redness and swelling of the tracheal and bronchial mucosa in the membranous lesion. Histologic examination of the bronchial biopsy showed inflammatory cell infiltration in the sub-mucosa with no vasculitis. Serum anti-type 2 collagen antibodies were found to be positive (33.9 EU/mL). Corticosteroid treatment improved his tracheochondritis. It is challenging to diagnose RP in the early stage due to its rarity and nonspecific symptoms. Airway involvement in RP is irreversible and the major cause of morbidity and mortality; hence, early recognition of airway involvement and treatment is warranted.

## Introduction

Relapsing polychondritis (RP) is a chronic and potentially life-threatening disease associated with inflammation in cartilaginous structures, particularly the ears, nose, joints, and respiratory tract. RP can also affect other tissues, such as the eyes, heart, blood vessels, and nervous system. It is considered a rare disease, with an estimated incidence of 0.7-3.5 per million per year [1-3]. Due to its rarity and nonspecific symptoms at the initial presentation, the diagnosis of RP may be delayed. Here, we report a case of RP with tracheobronchitis in the early stage.

## Case presentation

A 60-year-old Japanese man was referred to Hamanomachi Hospital for a mild elevation of C-reactive protein (CRP) and mild anemia without any symptoms. His comorbidity was well-controlled glaucoma with brimonidine, dorzolamide, and bimatoprost eye drops. His medical history included mild coronavirus disease 2019 one year earlier. He had an almost negligible smoking history. He had no specific family history. The patient was found to have mild CRP elevation (0.72 mg/dL) on a medical checkup. On upper and lower gastrointestinal examinations at a general practitioner, chronic gastritis and mild reflux gastritis were noted. After a month of examination by a general practitioner, no cause was identified. The patient was then referred to the Department of General Medicine, Hamanomachi Hospital, because of prolonged elevation of CRP (3.95 mg/dL) and anemia (hemoglobin 12.6 g/dL).

In the outpatient clinic, the patient was stable with the following vital signs: a body temperature of 36.2°C, oxygen saturation of 98% on room air, and respiratory rate of 16 breaths per minute. The initial physical examination did not reveal any obvious abnormality, with no rale on his chest, no skin rash, or joint swelling. Urinalysis was negative for both urine protein and urine occult blood. Blood examination revealed mild CRP elevation (0.78 mg/dL), lactate dehydrogenase (260 U/L), creatine phosphokinase (305 U/L), and aspartate aminotransferase (52 U/L). His white blood cell count (5,900/µL) and a percentage of eosinophils (2.3%), serum levels of iron (78 µg/dL), ferritin (95 ng/mL), and unsaturated iron-binding capacity (196 µg/dL) were within the normal range.

**Figure 1:**
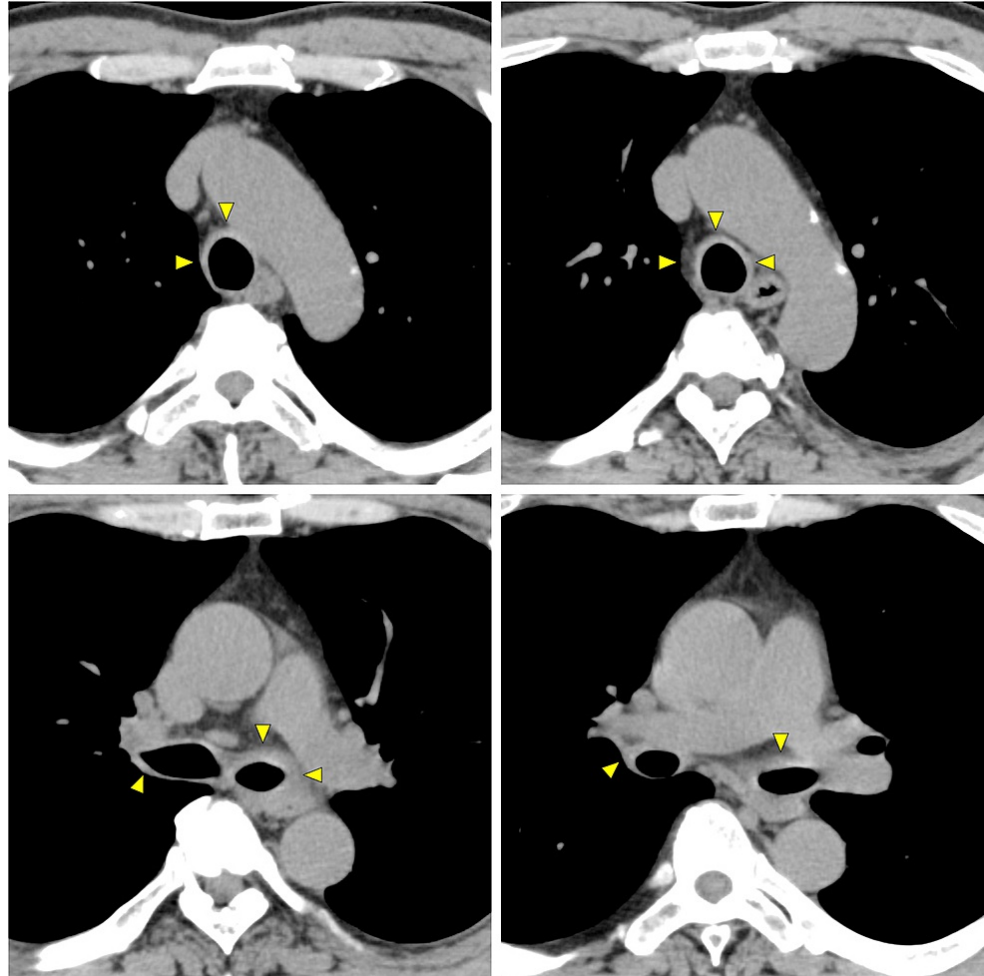
Chest CT images of the patient at the initial presentation. Thickened tracheal and bronchial walls (arrows) with no active lesions in the lung. There was no nodular wall thickening or gross calcification. There was a mild increase in the concentration of soft tissue around the trachea, with slightly enlarged mediastinal lymph nodes.

**Figure 2:**
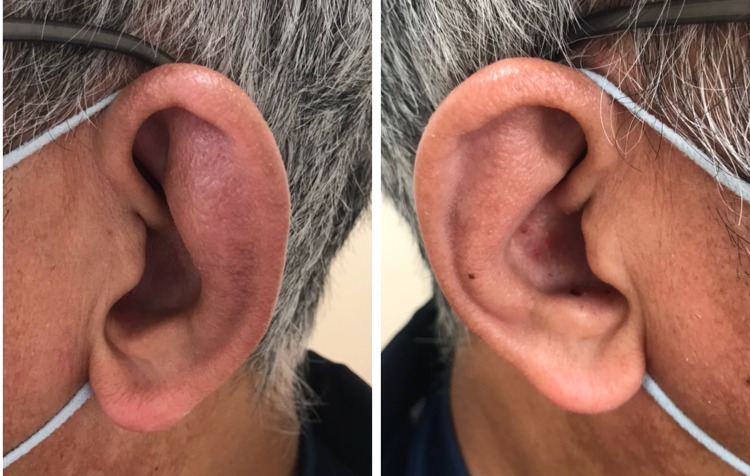
Bilateral auricular swelling at the first visit. The swelling was predominant in the left ear.

To investigate the possible source of the inflammation, body CT imaging was performed, which revealed thickened tracheal and bronchial walls with no active lesions in the lung (Figure 1). There was no nodular wall thickening or gross calcification. There was a mild increase in the concentration of soft tissue around the trachea, with slightly enlarged mediastinal lymph nodes. Differential diagnoses from the CT findings included eosinophilic granulomatosis with polyangiitis, amyloidosis, RP, tracheobronchopathia osteochondroplastica, and tracheal tuberculosis. The patient was then referred to the Department of Respiratory Medicine. Precise physical examination revealed bilateral ear swelling with no pain (Figure 2).

A CT imaging of the head confirmed bilateral auricular thickening but no specific findings in the nasal sinuses. The induced sputum test was negative for *Mycobacterium tuberculosis*. Additional blood examination was conducted, including erythrocyte sedimentation rate (ESR), the T-SPOT tuberculosis test, anti-neutrophil cytoplasmic antibodies, serum protein fractions, immunoglobulin (Ig) fractions with IgG4, anti-type 2 collagen antibodies (Table 1).

**Table 1:** Laboratory results on the first visit to Hamanomachi Hospital. CRP: C-reactive protein; ESR: erythrocyte sedimentation rate; IgG: immunoglobulin G; IgA: immunoglobulin A; IgM: immunoglobulin M; ANA: antinuclear antibody; RF: rheumatoid factor; CCP: cyclic citrullinated peptide; PR3-ANCA: proteinase-3-antineutrophil cytoplasmic antibody; MPO-ANCA: myeloperoxidase-antineutrophil cytoplasmic antibody

Test			Reference range
Blood test			
Hemoglobin	14.3	g/dL	13.7–16.8
CRP	0.78	mg/dL	0.0–0.14
ESR	38	mm/hour	0–10
IgG	1,684	mg/dL	861–1,747
IgA	248	mg/dL	93–393
IgM	70	mg/dL	33–183
IgG4	63.3	mg/dL	4.8–105
Protein fraction			
Alb	54.7	%	54.4–66.1
α1	3.7	%	2.7–4.3
α2	9.7	%	6.2–10.5
β	11.1	%	8.5–14.1
γ	20.8	%	12.3–22.8
ANA	<40		<40
RF	negative	IU/mL	negative
Anti-CCP antibody	1.2	U/mL	0.0–4.4
PR3-ANCA	<1.0	U/mL	0–3.4
MPO-ANCA	<1.0	U/mL	0–3.4
T-SPOT	Negative		Negative
Urinalysis			
pH	6.5		5–6
Specific gravity	1.013		
Protein	0	mg/dL	0
Red blood cells	0.9	/HPF	0
Cast	0	/LPF	0
Bence Jones protein	Negative		Negative

**Figure 3:**
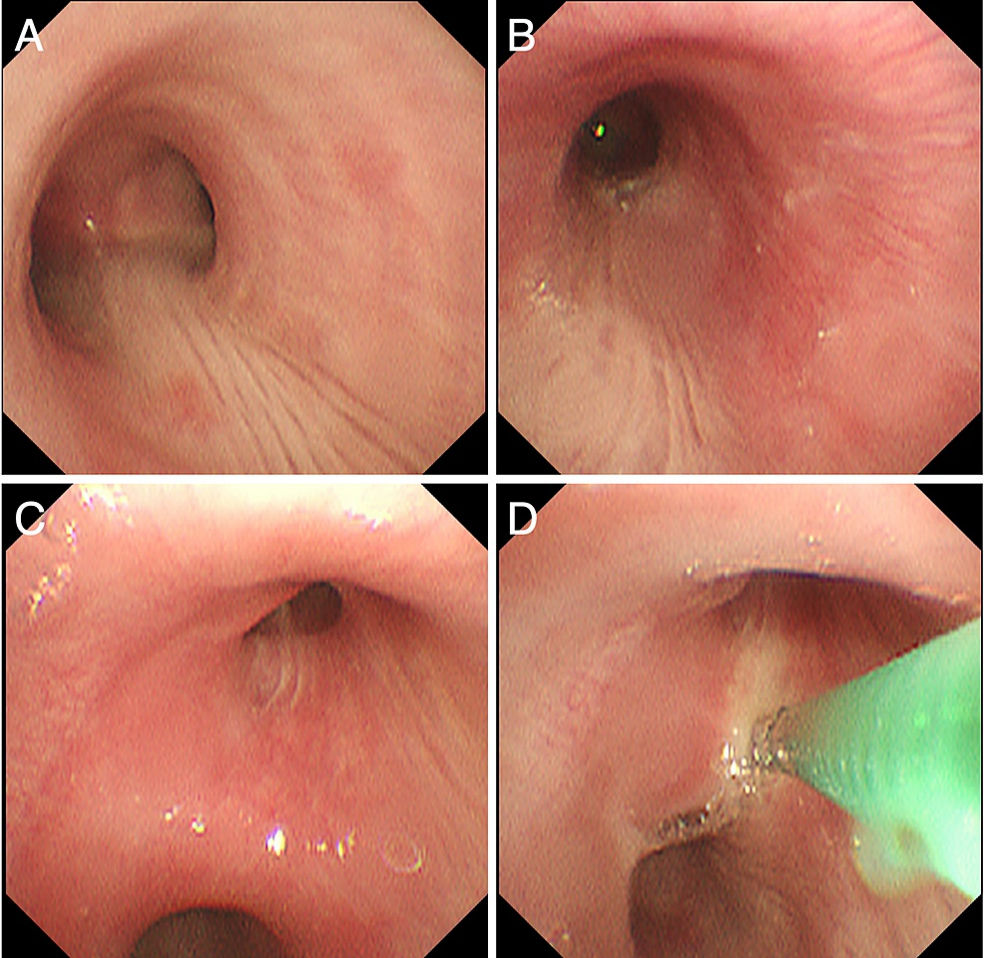
Bronchoscopic findings. Redness and swelling were mainly found in the tracheal and bronchial cartilage lesion, and the membranous lesion was almost normal. (A) Main carina. (B) The entrance of the left main bronchus. (C) The entrance of the right upper lobe bronchus. (D) Transbronchial biopsy from the spur of the right upper lobe bronchus.

**Figure 4:**
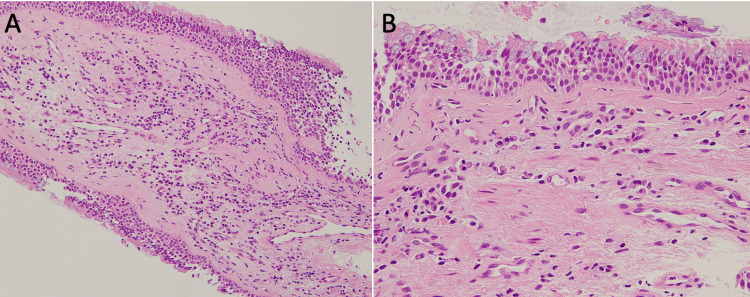
Pathological examination of the bronchial mucosa. (A, B) Hematoxylin and eosin staining of the biopsies from the spur of the right upper lobe bronchus. Inflammatory cell (lymphocyte predominant with some plasma cells and eosinophils) infiltration in the sub-mucosa with no vasculitis. There was no evidence of amyloidosis, infection, or malignancy. Magnification: (A) ×200, (B) ×400.

To confirm the diagnosis (especially to exclude other possible differential diagnoses such as tuberculosis, amyloidosis, and malignancy), a bronchoscopy was conducted. Grossly, redness and swelling were mainly found in the tracheal and bronchial cartilage lesion, and the membranous lesion was almost normal (Figure 3). Histologic examination of the bronchial biopsy showed inflammatory cell infiltration in the sub-mucosa with no vasculitis (Figure 4). There was no evidence of amyloidosis, infection, or malignancy. Serum anti-type 2 collagen antibodies were later found to be positive (33.9 EU/mL; normal range <20.0). Respiratory function revealed a slight decrease in peak expiratory flow (9.07 L/second, 91.5%).

**Figure 5:**
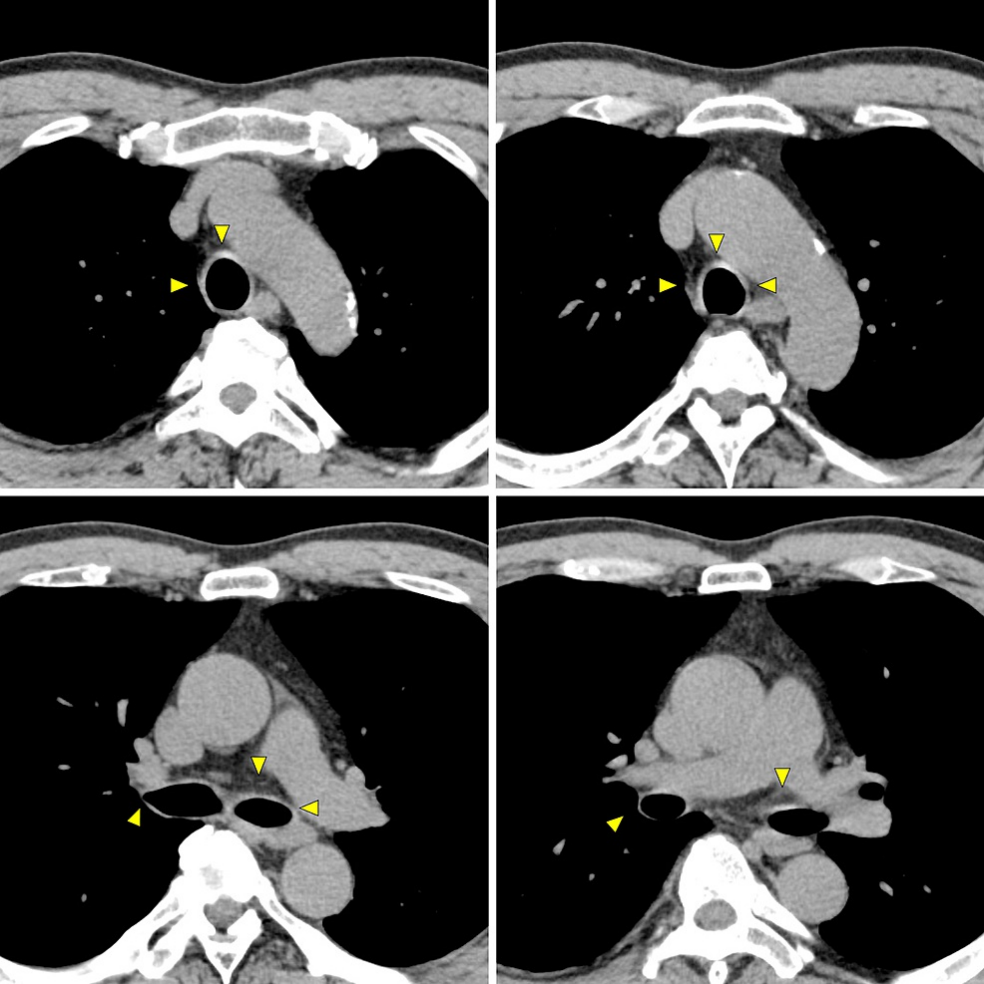
Follow-up chest CT images of the patient after treatment initiation. Improvement was found in the thickened tracheal and bronchial walls (arrows) and in the attenuation of soft tissue around the trachea.

Based on these results, RP was highly suspected, referring to the modified Damiani criteria [4] (Table 2). The patient was then initiated with 8 mg/day of methylprednisolone (mPSL) (10 mg of prednisolone equivalent) in the outpatient clinic for two weeks because of the difficulty for him to be hospitalized immediately. Although the levels of CRP and ESR normalized on admission to Hamanomachi Hospital, the ear swelling continued. The mPSL doses were then increased to 48 mg/day for one week after admission, followed by 16 mg/day. A follow-up CT of the chest one month after treatment initiation at discharge revealed improvement of his tracheochondritis (Figure 5). His ear swelling also improved at discharge. His clinical course confirmed the diagnosis of RP according to the modified Damiani criteria (Table 2).

**Table 2:** Diagnostic criteria for relapsing polychondritis.

Criteria	
McAdam’s criteria	
Required the presence of three or more of the following clinical features:	
(a) Bilateral auricular chondritis	
(b) Nonerosive seronegative inflammatory polyarthritis	
(c) Nasal chondritis	
(d) Ocular inflammation	
(e) Respiratory tract chondritis	
(f) Audiovestibular damage	
Modified Damiani criteria	
Required to have one of the following:	
(a) At least three of McAdam’s diagnostic criteria	
(b) One or more of McAdam’s findings with positive histologic confirmation	
(c) Chondritis at two or more separate anatomic locations with a response to treatment	

## Discussion

Disease progression in RP is generally slow but can result in irreversible organ damage during repeated relapses and remissions. In particular, airway involvement can lead to fatal outcomes [5], making early diagnosis necessary. However, early diagnosis of RP is often challenging. RP is defined using McAdam’s criteria (Table 2) [6], but only patients with advanced disease can meet the diagnostic criteria. Modifications to McAdam’s criteria (modified Damiamni criteria) (Table 2) were subsequently proposed to diagnose RP with variable clinical manifestations in the early period. The average time from onset to diagnosis has been reported to be 2.9 years, and approximately one-third of patients are reported to have visited more than five doctors before diagnosis [7]. Further, RP can be misdiagnosed as bronchial asthma [8]. There are several case reports where the patients had already developed tracheobronchomalacia at the time of the diagnosis of RP [9,10]. Thanks to Dr. Setoguchi and Dr. Sunami, professional radiologists, we were able to make differential diagnoses from the early CT findings in this case. Therefore, this case report suggests the importance of the radiologist’s role in the early diagnosis of RP. Precise physical examination revealed bilateral auricular swelling, which allowed us to suspect RP more strongly. The most common sign of RP is auricular inflammation, which accounts for 89% of patients with RP [1].

As summarized in a review article, there are several differential diagnoses for tracheal diseases [11]. On CT, RP is characterized by increased attenuation and thickening of the anterior and lateral walls of the large airways with sparing of the posterior wall. Tracheobronchial amyloidosis is characterized by diffuse nodular thickening of the trachea and main bronchi, commonly accompanied by nodular calcified regions within the trachea. Tracheobronchopatia osteochondroplastica is characterized by 1-3 mm nodules from the cartilaginous rings protruding into the airway lumen. Granulomatosis with polyangiitis is characterized by the diffuse or focally circumferentially thickened trachea and main bronchi. Peripheral bronchial narrowing, coexistent pulmonary nodules and masses, consolidation, and ground-glass opacities may also be present. Tracheobronchial tuberculosis shows circumferential and predominantly irregular luminal narrowing, accompanied by increased attenuation in the mediastinal fat. In this case, the CT findings were consistent with RP. Because no studies have described CT findings in the early stage of RP, this case report could be of use for clinicians.

Although the pathological findings of the bronchial biopsy helped support the diagnosis of RP and exclude other diseases, it was not possible to make a definitive diagnosis of RP because it did not include cartilaginous areas. The patient had an auricular swelling, and auricular cartilage biopsy would probably have led to a histologically definitive diagnosis of RP. However, when the results of the bronchial mucosal biopsy were obtained, we concluded that it was clinically consistent with RP and there were no significant changes in the treatment plan; hence, we did not perform further invasive tests.

The patient developed transient anemia before the diagnosis of RP. Anemia is usually present at some point during the course of RP [12]. It is typically normocytic and normochromic, consistent with anemia of chronic disease, as in this case.

We found elevated anti-type 2 collagen antibodies in the case. Its usefulness in evaluating the disease activity has been reported [13]. However, anti-type 2 collagen antibodies are found in less than half of the patients with RP. These antibodies can be detected in other diseases, including rheumatoid arthritis, seronegative spondyloarthropathies, systemic sclerosis, lupus, and Sjogren’s syndrome [13,14]. Further studies are warranted to evaluate anti-type 2 collagen antibodies as a biomarker for the response to therapy.

We initiated corticosteroid therapy at medium dosages to improve chondritis. In patients with mild-to-moderate condition with tracheal/bronchial involvement, prednisone at 1 mg/kg/day by mouth in divided doses for three to four weeks is generally recommended [15]. We, too, considered the same treatment dose and duration, but we chose lower doses based on the risk-benefit of corticosteroids and disease severity. In patients with more severe conditions with tracheal/bronchial involvement, systemic corticosteroids in combination with immunomodulatory agents can be administered, such as methotrexate, cyclosporine, azathioprine, and mycophenolate [16]. Biologic agents such as anti-tumor necrosis factor α (TNFα) (infliximab, etanercept, adalimumab), anti-interleukin (IL)-6 (tocilizumab), and anti-CD20 (rituximab) have also shown successful responses in steroid-refractory cases. The largest study from a French RP multicenter group reported 41 patients treated with 105 biologics [17]. The study found that TNF inhibitors, tocilizumab, and abatacept had the most significant impact on most cases of serious respiratory involvement [17]. However, there are no controlled trials involving these agents because of the rarity of the condition. These biologics have not been approved for the treatment of RP in countries such as Japan, the United States, and Europe. Further evidence is needed for understanding this rare autoimmune disease, and this case report may help recognize the early stage of the disease.

## Conclusions

We presented a case of RP with unexplained CRP elevation and mild anemia on a medical checkup that was later determined to be an early manifestation of RP. The patient had tracheobronchial and auricular chondritis without any symptoms. After the diagnosis, the patient was initiated on mPSL. Slight changes in the trachea on chest CT imaging helped make differential diagnoses, and precise physical examinations narrowed down the differential diagnosis. Early recognition and treatment of RP are warranted for a better prognosis, especially in patients with respiratory tract chondritis.
